# Delayed Tibial Osteomyelitis 37 Years After Habu Snake Envenomation: Limb Salvage With Local Antibiotic Perfusion and Areolar Tissue Grafting

**DOI:** 10.7759/cureus.90938

**Published:** 2025-08-25

**Authors:** Shohei Ishihara, Yusuke Shimizu, Edward H Ntege, Eri Yoshida

**Affiliations:** 1 Department of Plastic and Reconstructive Surgery, Graduate School of Medicine, University of the Ryukyus, Ginowan, JPN; 2 Department of Plastic and Reconstructive Surgery, Naha City Hospital, Naha, JPN

**Keywords:** antibiotic perfusion, chronic osteomyelitis, elderly patients, habu snake, limb salvage, perifascial areolar tissue, snakebite, staphylococcus lugdunensis, tibial reconstruction

## Abstract

Chronic osteomyelitis in elderly patients traditionally necessitates extensive surgical interventions associated with high morbidity. We report successful minimally invasive limb salvage in an 84-year-old man with chronic tibial osteomyelitis, employing continuous local antibiotic perfusion (CLAP) and perifascial areolar tissue (PAT) grafting. The patient presented with a one-year history of purulent drainage from the anterior tibial crest, occurring 37 years after a habu snake (*Protobothrops flavoviridis*) bite at the same site. MRI revealed characteristic bone marrow edema, and microbiological analysis identified *Staphylococcus lugdunensis*. Following surgical debridement, the CLAP system delivered gentamicin at 1,200 µg/mL through stainless-steel intramedullary antibiotic perfusion pins for 14 days. Reconstruction was completed using a PAT flap harvested from the external oblique fascia, followed by split-thickness skin grafting. The patient achieved complete wound healing, pain-free ambulation, and excellent functional recovery at six-month follow-up without complications. This case highlights the efficacy of modern minimally invasive techniques for managing complex bone infections in elderly patients. The combination of targeted local antibiotic delivery and low-morbidity tissue reconstruction achieved limb salvage while minimizing surgical risks, suggesting broader applicability in similarly vulnerable populations.

## Introduction

Snakebite envenomation remains a significant global public health concern, with an estimated 1.8 to 2.7 million cases of envenoming each year, predominantly in tropical and subtropical regions [1]. The burden is highest in South/Southeast Asia and sub-Saharan Africa. In Japan, the habu snake (*Protobothrops flavoviridis*) is the most medically important viperid in Okinawa Prefecture and its surrounding islands, with approximately 100 habu bites and 1,000 mamushi bites annually [2,3]. Its venom is rich in snake-venom metalloproteinases and other enzymes that facilitate tissue invasion (e.g., hyaluronidase), enabling deep-tissue spread and potential bacterial inoculation [4,5]. Reported habu-bite incidence in Okinawa has declined over recent decades, with current summaries noting approximately 50 to 100 cases per year; however, the clinical impact remains substantial [6,7].

Acute complications of habu envenomation, such as local necrosis, hemorrhage, and compartment syndrome requiring fasciotomy, are well documented. These effects primarily result from venom-derived enzymes that induce tissue destruction and facilitate deep venom diffusion [6]. Given the substantial fang length of the habu snake, intramuscular venom injection capable of reaching bone is plausible, particularly in anatomical regions with minimal soft-tissue coverage. Despite this, bacterial infections following habu bites have rarely been reported, making delayed infectious complications such as osteomyelitis exceedingly uncommon.

Chronic osteomyelitis poses significant diagnostic and therapeutic challenges, particularly among older adults with comorbidities. Characterized by persistent infection, biofilm formation, and limited antibiotic penetration, it contributes to substantial morbidity and complexity of care. In developed countries, including the United States and Japan, it represents a meaningful healthcare burden [8]. *Staphylococcus aureus*, the most common causative organism, is known for its ability to persist intracellularly within osteoblasts, enabling dormancy and reactivation even decades later [9].

Managing chronic osteomyelitis in older patients is particularly challenging, as conventional treatment often entails extensive debridement and complex flap reconstruction, procedures associated with substantial perioperative risk. To address these limitations, advances in targeted antibiotic delivery and simplified tissue reconstruction have emerged as promising alternatives. Continuous local antibiotic perfusion (CLAP), a drug-delivery system, not a standalone therapy, delivers antibiotics directly into infected medullary cavities through intramedullary antibiotic perfusion (iMAP) pins [10]. This approach sustains intraosseous concentrations that exceed minimum biofilm-eradication thresholds while maintaining low systemic exposure. For example, in a prospective series of 40 patients, Maruo et al. [11] achieved median local gentamicin levels >500 µg/mL while keeping mean serum troughs <2 µg/mL at a perfusion concentration of 1.2 mg/mL, demonstrating a favorable therapeutic index without clinically significant nephrotoxicity or ototoxicity. Similarly, perifascial areolar tissue (PAT) grafting has emerged as a low-morbidity reconstructive option for covering exposed bone in patients who are unsuitable for extensive flap procedures [12,13].

Osteomyelitis following animal bites, particularly snakebites, is rare. A recent systematic review found a roughly 27% prevalence of snakebite-associated wound infections overall, yet osteomyelitis remains confined to case reports, supporting its characterization as a rare event [14]. Delayed osteomyelitis, manifesting weeks to decades after envenomation, has been sporadically reported, including *Proteus* species osteomyelitis five weeks post bite and a *Staphylococcus aureus* case reactivating 75 years after presumed initial inoculation [15,16]. Anatomical regions with minimal soft-tissue coverage, such as the anterior tibial crest, may be especially prone to deep bacterial inoculation during penetrating trauma like snakebites, given the fang length and venom diffusion capability of the habu snake.

Here, we report a case of delayed tibial osteomyelitis developing an extraordinary 37 years after a habu snakebite, representing an exceptionally long latency period for osteomyelitis following habu envenomation. The patient, an older adult with no intervening trauma, presented with a draining sinus precisely at the original bite site. He was successfully treated using a minimally invasive strategy combining targeted (minimal) debridement supported by the CLAP drug-delivery system and PAT grafting. This case demonstrates the potential of contemporary, targeted approaches to achieve durable infection control and functional preservation in high-risk patients. Furthermore, this case underscores several key clinical lessons: the importance of recognizing extremely delayed infectious sequelae following penetrating injury; the applicability of low-morbidity reconstructive options in older adults; and the value of modern, localized therapeutic strategies as viable alternatives to traditional, high-risk reconstruction in select patient populations.

## Case presentation

An 84-year-old Japanese man presented to our plastic and reconstructive surgery department with a one-year history of chronic purulent drainage and a non-healing ulcer on the anterior aspect of his right lower leg. His medical history included well-controlled hypertension and dyslipidemia. Notably, he reported having been bitten by a habu snake at the same anatomical location in 1987. However, no contemporaneous 1987 treatment records were available, and the species identification and wound location are based on patient recall. At that time, he reportedly received antivenom and supportive care at a local hospital, with complete wound healing within several weeks and no documented complications. He denied any subsequent trauma, surgery, or injury to the affected limb during the 37 years that followed.

Over the preceding year, an ulcer gradually developed at the previously healed bite site without intervening trauma. It progressed to a chronic draining sinus with intermittent purulent discharge, refractory to conservative wound care and empiric oral antibiotics from a local clinic. The patient reported mild activity-related pain (numerical rating scale (NRS) 0-10, score 2/10) and denied fever, weight loss, or other systemic symptoms.

**Figure 1:**
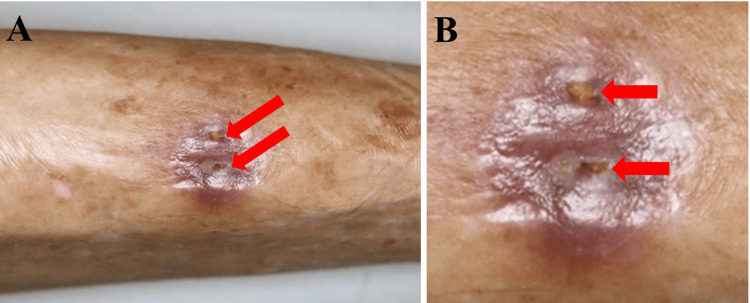
Preoperative clinical presentation of the anterior tibia (A) Overview photograph showing two cutaneous sinus tract openings aligned over the tibial crest (red arrows); (B) Magnified view highlighting the two sinus tract orifices (red arrows) with punctate yellow exudate; note the surrounding post-inflammatory hyperpigmentation and mature scarring. The original 1987 bite scar cannot be identified in this image.

Physical examination revealed an elderly man in no acute distress with stable vital signs. Two distinct cutaneous fistulas were noted on the anterior tibial crest, approximately 15 cm distal to the tibial tuberosity (Figure 1). Each opening measured approximately 3-4 mm in diameter and was actively draining purulent material. The surrounding skin displayed mature fibrous scar tissue and hyperpigmentation consistent with chronic post-traumatic changes. The tibia was easily palpable beneath minimal soft tissue coverage, and subtle contour deformity suggested underlying bone involvement. Mild tenderness was elicited around the fistulous tracts, without fluctuance or surrounding erythema. Distal pulses (dorsalis pedis and posterior tibial) were palpable, with intact sensation and full range of motion at the ankle and subtalar joints.

A comprehensive laboratory evaluation was performed preoperatively and serially postoperatively; key results are summarized in Table 1. Baseline studies demonstrated mild anemia (hemoglobin: 9.9 g/dL) consistent with chronic disease, a normal white-blood-cell count (5.0 × 10³/µL) indicating absence of systemic infection, and a very low C-reactive protein (0.023 mg/dL), all of which supported a chronic rather than acute inflammatory process. Nutritional and metabolic parameters were within normal limits (serum albumin: 3.9 g/dL; hemoglobin A1c: 5.5%), and HIV testing was negative.

**Table 1:** Serial laboratory investigations (preoperative and postoperative day (POD) time points) † POD 11 values are shown only for tests routinely repeated at that visit; ND indicates the test was not measured on that day. L: below reference range; Serum gentamicin troughs at POD 3 = 0.62 µg/mL and POD 10 = 0.41 µg/mL remained well below nephrotoxic thresholds; renal function was stable throughout.

Parameter	Pre-op	POD 3	POD 10	POD 11†	Reference range	Units
White blood cells (WBC)	5.0	ND	ND	5.1	4.0–10.0	×10³/µL
Red blood cells	3.36 (L)	ND	ND	3.08 (L)	4.2–5.4	×10⁶/µL
Hemoglobin	9.9 (L)	ND	ND	9.8 (L)	13.5–17.5	g/dL
Hematocrit	29.7 (L)	ND	ND	30.3 (L)	41–50	%
Platelet count	15.2	ND	ND	24.1	15–45	×10⁴/µL
C-reactive protein (CRP)	0.023	ND	0.259	ND	<0.3	mg/dL
Creatinine	0.82	0.80	0.79	ND	0.60–1.20	mg/dL
Serum gentamicin (GM)	ND	0.62	0.41	ND	<2.0	µg/mL
Serum albumin	3.9	ND	ND	ND	3.5–5.0	g/dL
Hemoglobin A1c	5.5	ND	ND	ND	<6.5	%

To monitor systemic exposure to the locally perfused gentamicin, serum trough levels were obtained on postoperative day (POD) 3 and POD 10, measuring 0.62 µg/mL and 0.41 µg/mL, respectively, both well below the nephrotoxic threshold of < 2 µg/mL. No clinically significant trends were observed in leukocyte count, renal function, or inflammatory markers during follow-up.

**Figure 2:**
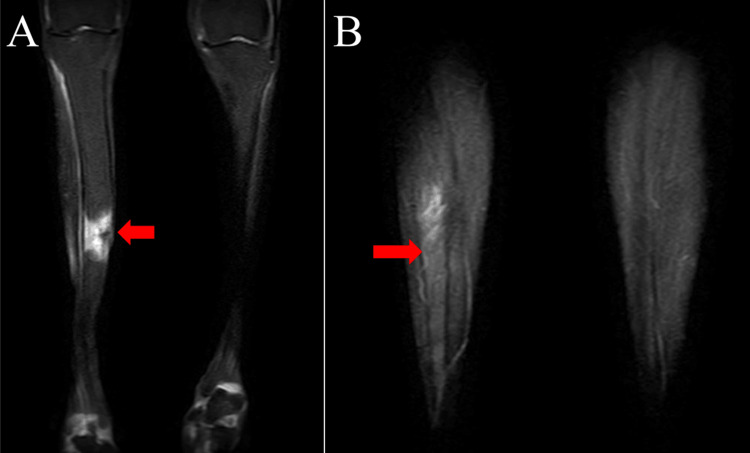
Preoperative short-tau inversion-recovery (STIR)-MRI findings (A) Coronal STIR image showing a high STIR area in the anterior tibial cortex and medullary cavity (red arrowhead), consistent with marrow edema and inflammation; (B) Axial STIR image demonstrating cortical irregularity with matching medullary hyperintensity precisely at the historical snakebite site (red arrowhead).

The diagnostic workup prioritized ruling out malignancy and determining the infectious etiology through a multimodal approach. Short-tau inversion-recovery (STIR) MRI demonstrated a high STIR area in the anterior cortex and medullary cavity of the tibia directly beneath the cutaneous fistulas (Figures 2A, 2B), reflecting marrow edema and inflammation. This signal alteration corresponded precisely to the historical snakebite site, providing critical anatomical correlation.

Deep incisional biopsies of the ulcer edge and sinus-tract wall demonstrated hyperkeratotic epidermis with pseudo-epitheliomatous hyperplasia overlying dense chronic inflammatory granulation tissue; no epithelial dysplasia or invasive squamous-cell carcinoma was seen on hematoxylin and eosin staining or on ΔNp63 (p40), p63 (TP63), and cytokeratin 5/6 (CK5/6) immunohistochemistry. Intraoperative bone culture yielded methicillin-susceptible *Staphylococcus lugdunensis* (gentamicin minimum inhibitory concentration (MIC) ≤ 1 µg/mL, susceptible to first-generation cephalosporins). Together with the MRI showing a high STIR signal within the tibial medullary cavity, these findings confirmed the diagnosis of chronic osteomyelitis.

Guided by the susceptibility profile, the patient received intravenous cefazolin 1 g every eight hours for the first 72 hours (POD 0 to POD 3), followed by oral cefaclor 250 mg three times daily for six weeks. This systemic regimen complemented the high-dose local gentamicin perfusion delivered via the CLAP system. The microbiological findings are shown in Table 2, and the complete local-versus-systemic antibiotic strategy is summarized in Table 3.

**Table 2:** Deep-bone culture and systemic susceptibility profile Microbiological profile of deep medullary bone obtained intra-operatively after cortical windowing (no superficial swabs). Breakpoints reflect systemic criteria of the Clinical and Laboratory Standards Institute (CLSI) [17] and the European Committee on Antimicrobial Susceptibility Testing (EUCAST) [18]. These interpretive categories guide systemic antibiotic choice only. Continuous local antibiotic perfusion (CLAP) achieves intramedullary gentamicin concentrations >500 µg/mL at 1.2 mg/mL perfusion, so systemic breakpoints do not predict local efficacy; local effect depends on the local concentration: minimum inhibitory concentration (MIC) ratio; *Cefaclor selection was inferred from cefazolin susceptibility/class effect as the oral step-down agent.

Antibiotic (route)	MIC (µg/mL)	Interpretation (CLSI/EUCAST)	Clinical relevance
Penicillin G	>4	Resistant	Typical resistance for *Staphylococcus* *lugdunensis*
Cefazolin (IV)	≤4	Susceptible	Selected for initial systemic coverage (postoperative day (POD) 0 to POD 3)
Cefaclor (oral)*	NT	—	Not tested; selected as oral step-down based on first-generation cephalosporin susceptibility (cefazolin) and methicillin-susceptible phenotype
Gentamicin	≤1	Susceptible	Aminoglycosides active in vitro; used locally via CLAP (pharmacokinetics exceed serum breakpoints)
Amikacin	≤1	Susceptible	Aminoglycoside class alternative (not used)
Imipenem	≤0.25	Susceptible	Broad-spectrum reserve (not used)
Minocycline	≤2	Susceptible	Oral alternative (not used)

**Table 3:** Local (continuous local antibiotic perfusion (CLAP)) vs systemic antibiotic regimens and monitoring iMAP: intramedullary antibiotic perfusion; POD: postoperative day; MSSA: methicillin-susceptible *Staphylococcus aureus* (susceptible to antistaphylococcal β-lactams and first-generation cephalosporins). Serum gentamicin values reflect systemic absorption of locally perfused drug and remained below typical nephrotoxic thresholds (<2 µg/mL). Interpretive minimum inhibitory concentration (MIC) breakpoints (the Clinical and Laboratory Standards Institute (CLSI) [17] and the European Committee on Antimicrobial Susceptibility Testing (EUCAST) [18] apply to serum/systemic therapy and do not predict local continuous local antibiotic perfusion (CLAP) efficacy; local effect depends on achieving very high local concentration of MIC ratios.

Route	Agent (formulation)	Dose/concentration	Delivery mode and window	Duration	Key monitoring results	Purpose
Local	Gentamicin solution (1.2 mg/mL ≈ 1,200 µg/mL)	2 mL/h, continuous	CLAP via two intramedullary iMAP pins (POD 0–14)	14 days	Serum gentamicin (spillover): POD 3 = 0.62 µg/mL; POD 10 = 0.41 µg/mL; creatinine stable	High intramedullary bactericidal levels (>500 µg/mL reported with 1.2 mg/mL perfusion); biofilm-active
Systemic: phase 1	Cefazolin IV	1 g every 8 hours	Inpatient (POD 0–3)	72 hours	Creatinine remained within normal limits; no adverse events	Immediate bactericidal cover for MSSA/ *Staphylococcus lugdunensi*s after debridement
Systemic: phase 2	Cefaclor PO	250 mg three times daily	Outpatient (POD 4–45)	6 weeks	Creatinine stable; clinical response maintained	Oral step-down maintaining the first-generation cephalosporin spectrum

The differential diagnosis included squamous-cell carcinoma (Marjolin ulcer), metastatic bone disease, atypical mycobacterial infection, and actinomycosis. An integrated assessment MRI demonstrating a high STIR area beneath the sinus tracts, deep bone culture growing methicillin-susceptible *Staphylococcus lugdunensis*, and histology/immunohistochemistry excluding malignancy-together with precise anatomical concordance with the historical snakebite site, was most consistent with chronic osteomyelitis, likely representing reactivation at the original inoculation site (Table 4).

**Table 4:** Diagnostic components supporting the final diagnosis of chronic tibial osteomyelitis NRS: numerical rating scale; STIR: short tau inversion recovery; iMAP: intramedullary antibiotic perfusion; CLAP: continuous local antibiotic perfusion; SCC: squamous-cell carcinoma; IHC: immunohistochemistry. Imaging wording uses the neutral descriptor “high STIR area” to avoid equating the entire hyperintense region with active infection. Cultures were obtained from deep bone intraoperatively (no superficial swabs). Normal CRP/WBC does not exclude chronic osteomyelitis; sensitivity is limited once infection is compartmentalized.

Component	Evidence in this case	Role in diagnosis
Clinical features	One-year history of a chronic draining sinus with intermittent purulence at the prior bite site; mild activity-related pain (NRS 2/10); no fever or systemic symptoms.	Primary: classic presentation of chronic osteomyelitis with sinus formation.
Anatomical correlation	Lesion located precisely at the historical habu snakebite site on the anterior tibial crest (minimal soft-tissue coverage).	Supportive: plausible portal for deep inoculation/reactivation.
Imaging (MRI, STIR)	High STIR area in the anterior cortex and medullary cavity directly beneath the cutaneous fistulas (Figures 2A–B).	Supportive: maps marrow edema/inflammation; guided iMAP placement.
Microbiology (deep-bone culture)	Intra-operative deep medullary bone culture: methicillin-susceptible Staphylococcus lugdunensis (cefazolin susceptible; gentamicin MIC ≤1 µg/mL).	Primary: organism identified from deep tissue confirms infection.
Histopathology/malignancy exclusion	Sinus-edge and tract biopsies: chronic inflammatory infiltrate; no malignancy. IHC (p40/p63/CK5/6) negative, favoring reactive pseudo-epitheliomatous hyperplasia.	Exclusionary/Supportive: rules out Marjolin (SCC); supports chronic infection.
Laboratory markers	WBC 5.0 ×10³/µL, CRP 0.023 mg/dL at baseline; values remained low during follow-up.	Contextual: normal inflammatory markers are common in chronic disease; low sensitivity.
Therapeutic response (follow-up)	After targeted debridement plus CLAP and systemic antibiotics, the sinus closed and pain resolved (NRS 0/10); PAT graft remained intact at 6 months.	Supportive: clinical resolution consistent with infection control.

Given the patient’s advanced age, comorbidities, and localized infection, the treatment strategy prioritized minimally invasive options tailored to his physiologic reserve. Traditional management requiring extensive resection and microvascular reconstruction would have posed considerable perioperative risk. The goal was to achieve infection control and durable soft‑tissue coverage while minimizing surgical morbidity.

The CLAP drug‑delivery system was selected to deliver high‑concentration, site‑specific antibiotics while limiting systemic exposure. This approach achieves local drug concentrations more than 50‑fold higher than systemic exposure-relevant for biofilm control because CLAP at 1.2 mg/mL delivers intramedullary gentamicin ≥ 500 µg/mL with serum troughs typically < 2 µg/mL (our patient: 0.62 and 0.41 µg/mL; Table 3) [11, 19]. This aligns with established principles for chronic osteomyelitis management following adequate debridement [20].

**Figure 3:**
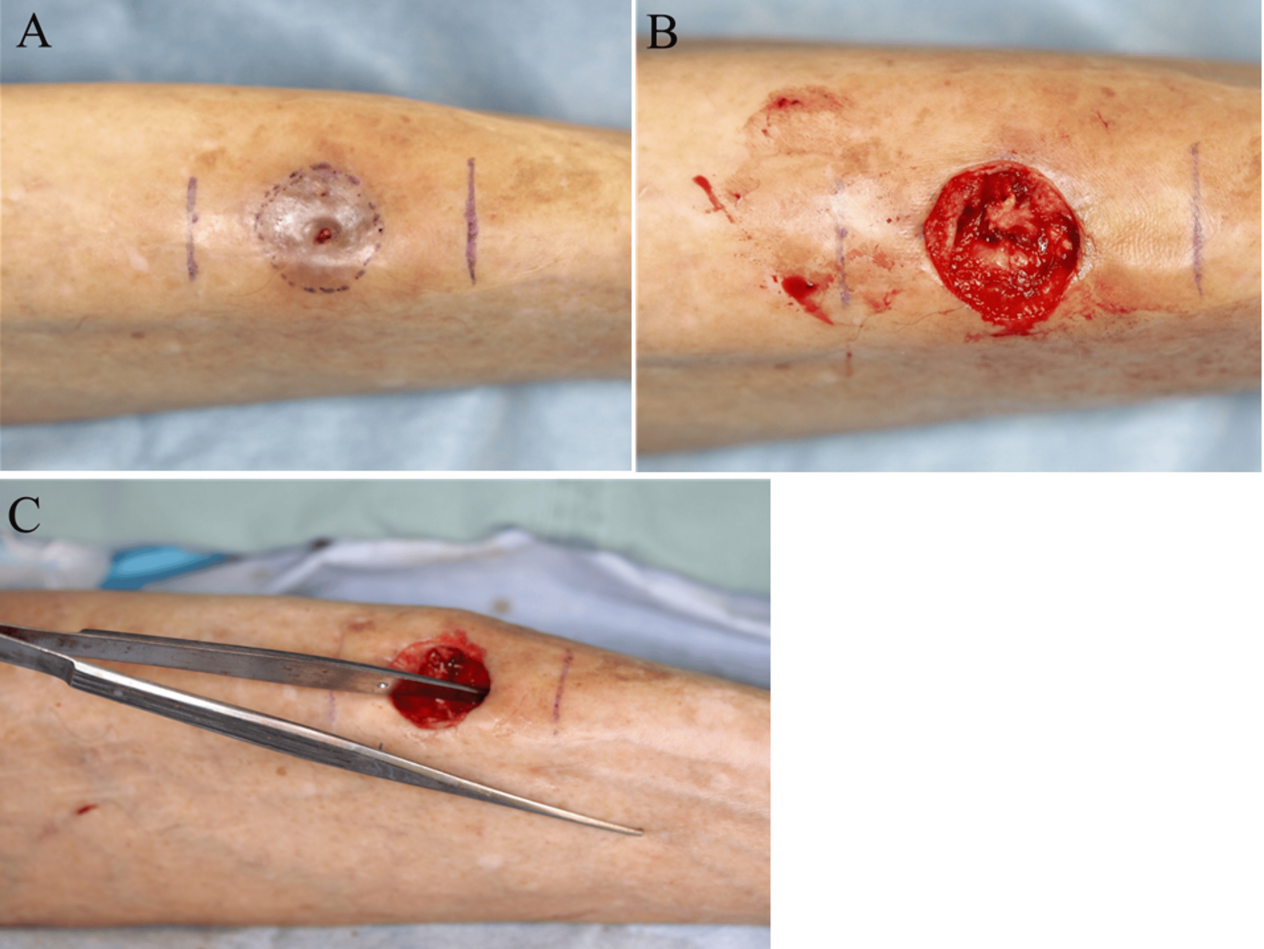
Intraoperative surgical findings (A) Elliptical skin excision encompassing both fistulous openings and the surrounding affected tissue; (B) Exposed anterior tibial cortex with multiple cortical defects and deformed bone; (C) Probing confirms deep medullary cavity involvement through cortical breaches.

The patient underwent surgical exploration under general anesthesia. An elliptical skin incision encompassed both fistulous openings, extending into healthy surrounding tissue (Figure 3A). Subperiosteal dissection exposed the anterior tibial cortex, revealing multiple cortical defects and irregular, deformed bone architecture (Figure 3B). Probing confirmed deep medullary extension of infection through the cortical defects (Figure 3C).

**Figure 4:**
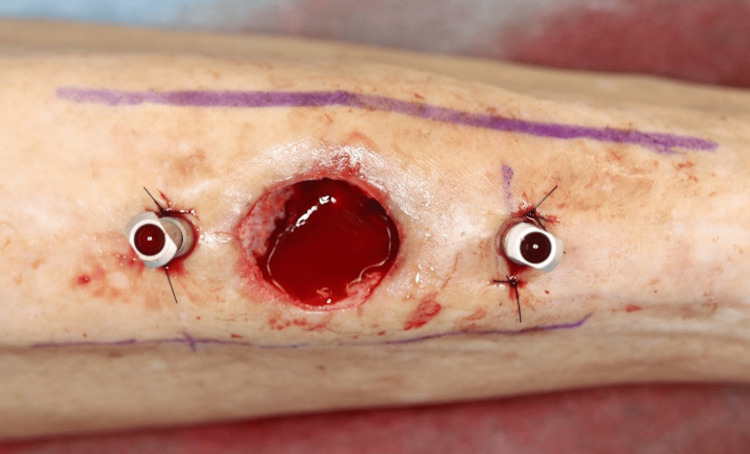
Placement of intramedullary antibiotic perfusion (iMAP) pins Bilateral iMAP pins positioned at the proximal and distal margins of infection, as defined by preoperative short-tau inversion-recovery (STIR)-MRI imaging.

Systematic intramedullary curettage was performed with rongeurs and curettes until healthy, bleeding bone was encountered. After the anterior cortical window was opened, a fragment of devitalized cancellous bone from the medullary cavity was lifted with a sterile curette, placed in a sealed container, and sent for aerobic and anaerobic culture; no superficial swabs were taken. Once thorough debridement was complete, two stainless-steel intramedullary antibiotic perfusion (iMAP) pins were positioned at the proximal and distal margins of the high STIR area identified on preoperative STIR-MRI to ensure uniform antibiotic distribution (Figure 4).

**Figure 5:**
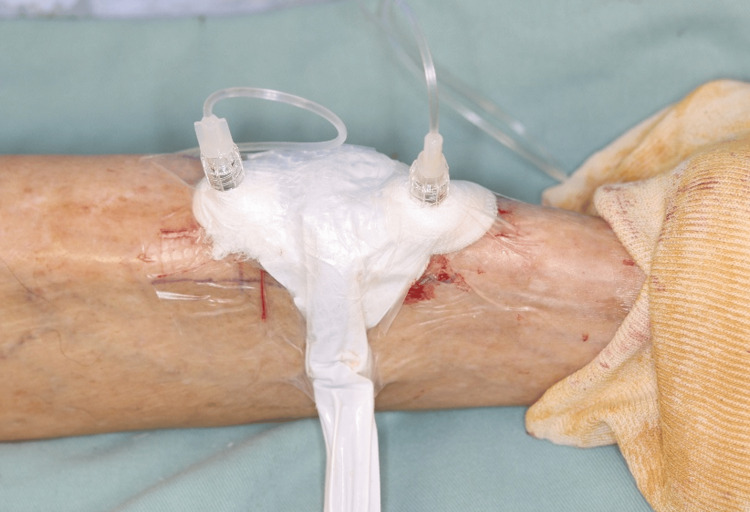
Postoperative negative-pressure wound therapy (NPWT) application The NPWT system was applied to promote granulation tissue and maintain a favourable wound environment during continuous local antibiotic perfusion (CLAP) delivery.

CLAP was initiated immediately postoperatively, delivering gentamicin at 1.2 mg/mL through two intramedullary iMAP pins at 2 mL/h. Antibiotic selection was guided by culture sensitivity testing. Negative-pressure wound therapy (NPWT) was applied concurrently to promote granulation tissue formation and maintain optimal wound conditions (Figure 5). CLAP continued for 14 days. Serum gentamicin levels were monitored at 0.62 µg/mL on POD 3 and 0.41 µg/mL on POD 10, mirroring the systemic values reported by Maruo et al. [14] and remaining well below nephrotoxic thresholds. Renal-function tests and daily subjective auditory assessments revealed no adverse effects, and all safety parameters stayed within acceptable limits.

**Figure 6:**
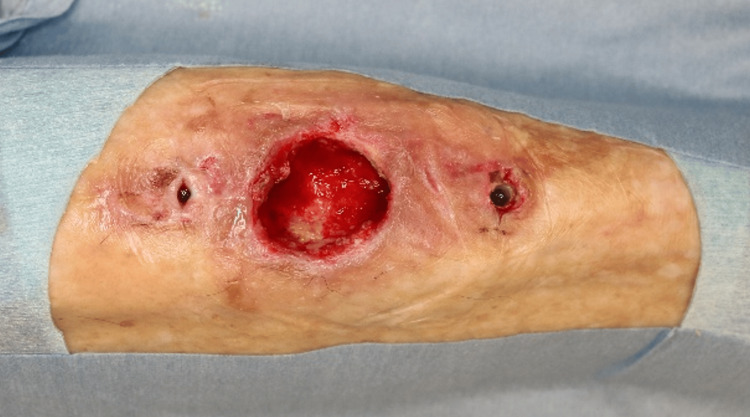
Wound status post continuous local antibiotic perfusion (CLAP) therapy Healthy, well-vascularized granulation tissue following 14 days of local antibiotic perfusion, indicating readiness for definitive reconstruction, and confirming that the wound bed met the prerequisites for perifascial areolar tissue (PAT) graft acceptance.

**Figure 7:**
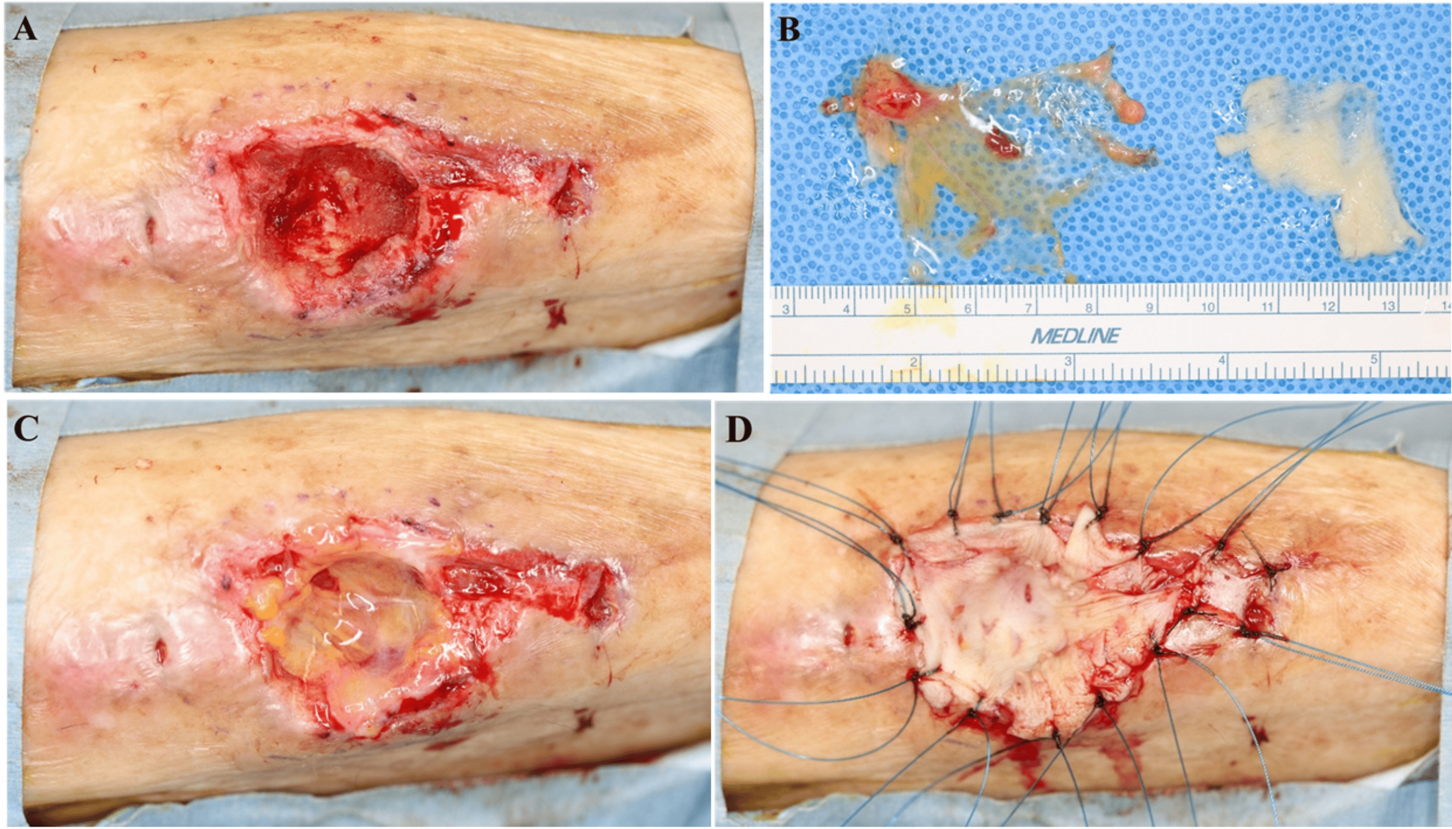
Sequence of soft tissue reconstruction (A) Granulation bed after continuous local antibiotic perfusion (CLAP) delivery and negative-pressure wound therapy (NPWT); (B) Harvested a 5 × 5 cm PAT flap and split-thickness skin graft; (C) Perifascial areolar tissue (PAT) flap inset into the debrided wound. (D) Skin graft secured with sutures and bolster dressing.

Following completion of the CLAP delivery system and confirmation of infection control, evidenced by robust granulation tissue formation (Figure 6), reconstruction proceeded. A 5 × 5 cm PAT flap was harvested from the external oblique aponeurosis of the lower abdomen (Figures 7A, 7B) and inset into the wound bed to provide vascularized soft tissue coverage (Figure 7C). A split-thickness skin graft (10/1000 inch) was subsequently applied over the PAT flap and secured with sutures and a bolster dressing (Figure 7D).

The patient tolerated all procedures without complication. Protected weight-bearing was initiated on postoperative day 10. The patient reported complete pain resolution, with an NRS pain score of 0. Satisfaction with both functional and cosmetic outcomes was high.

**Figure 8:**
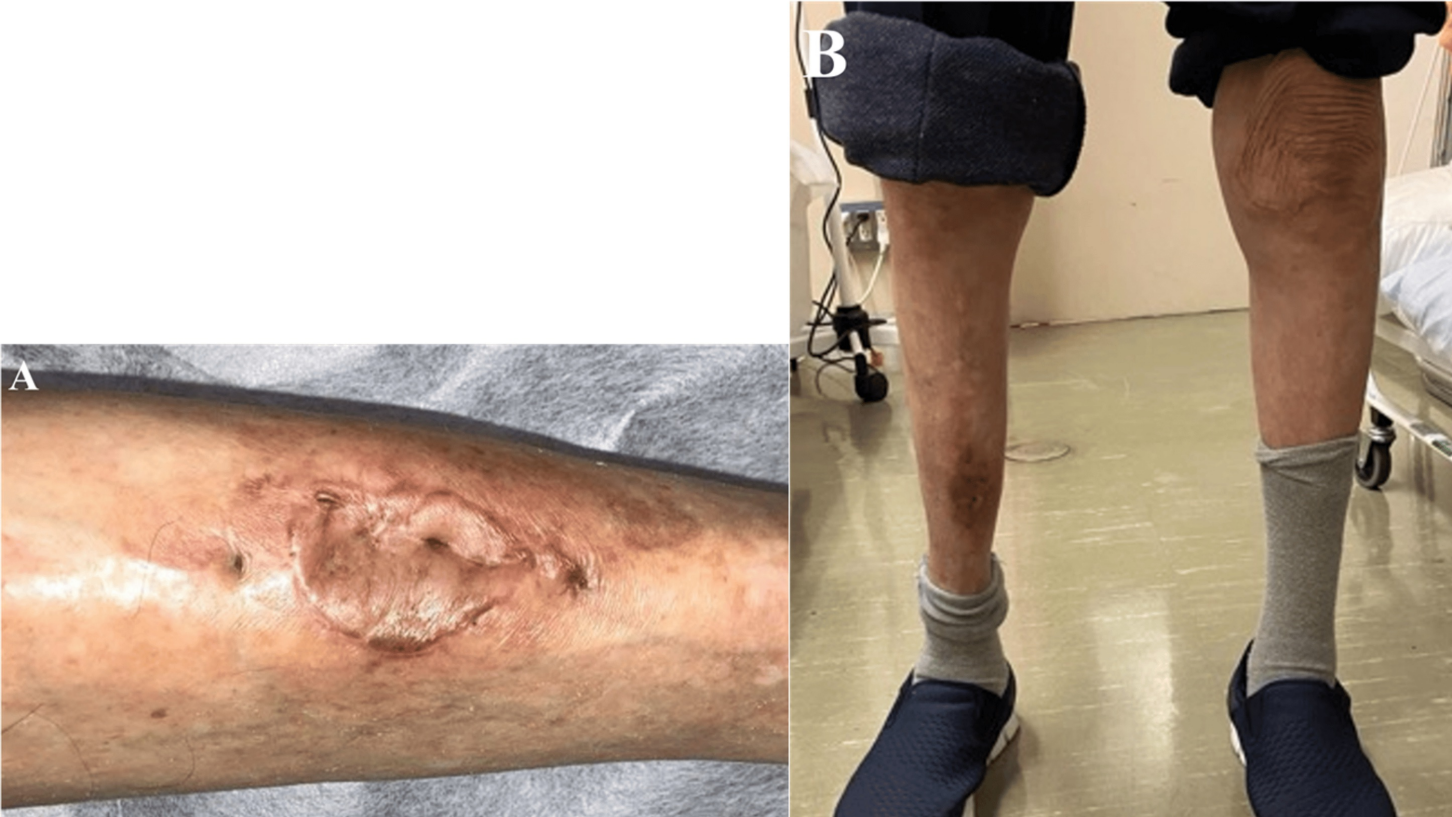
Six-month postoperative outcomes (A) Complete wound healing with excellent tissue integration and no signs of recurrence; (B) Independent ambulation with normal gait and full functional recovery.

**Figure 9:**
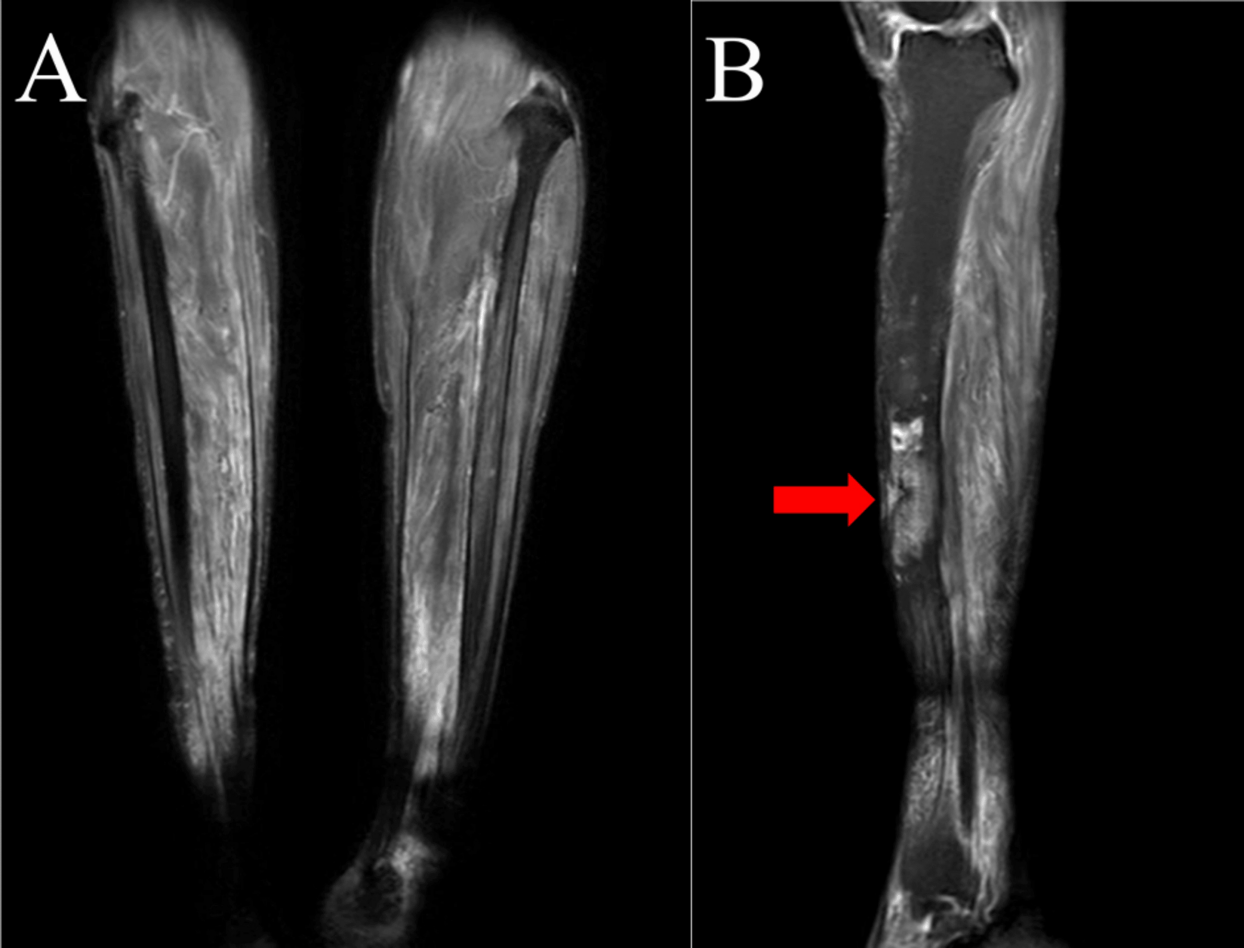
Two-month postoperative short-tau inversion-recovery (STIR) MRI findings (A) Coronal view shows resolution of preoperative signal abnormalities, consistent with bone healing; (B) Axial view confirms absence of cortical disruption or active inflammation (red arrowhead).

At six-month follow-up, the surgical site demonstrated complete epithelialization without recurrence of drainage, ulceration, or sinus formation (Figure 8A). The transplanted tissues were well integrated, with preserved contour and no signs of graft failure. Functional evaluation confirmed independent ambulation with normal gait mechanics and full return to baseline daily activity (Figure 8B). MRI-STIR imaging at two months postoperatively showed resolution of bone marrow edema and no evidence of active osteomyelitis (Figure 9).

## Discussion

This case demonstrates the successful application of contemporary, minimally invasive techniques for managing complex, chronic osteomyelitis in an older adult. While the exceptionally long 37‑year latency following habu envenomation is noteworthy, the greater significance lies in the treatment sequence used. Definitive infection control was achieved through targeted minimal debridement, followed by adjunctive delivery of high‑dose intramedullary gentamicin via the CLAP drug‑delivery system (through iMAP pins) to maintain a high local antibiotic gradient after mechanical source control. The defect was then covered with a PAT graft. This integrated approach achieved infection control and limb salvage while minimizing the morbidity typically associated with extensive bone resection and complex microvascular reconstruction.

The integration of the CLAP drug‑delivery system and PAT grafting represents a viable, low‑morbidity strategy for chronic bone infection in older adults. Traditional approaches involving radical debridement and microsurgical tissue transfer can carry higher perioperative risk and longer recovery in patients with comorbidities, with increased medical complication rates reported in large elderly cohorts [21]. In contrast, our approach achieved comparable infection eradication and soft‑tissue coverage through site‑specific antimicrobial delivery and low‑morbidity reconstruction [10, 22]. These outcomes suggest practical applicability for high‑risk populations who may not tolerate extensive procedures.

The CLAP delivery system delivers gentamicin at 1.2 mg/mL directly into the infected medullary cavity, maintaining high local concentrations while minimizing systemic exposure, an important consideration in older adults vulnerable to nephrotoxicity and ototoxicity [10, 11]. In a 40‑patient series, Maruo et al. [11] recorded median intramedullary levels > 500 µg/mL with serum troughs < 2 µg/mL and only a single transient renal event. Our patient’s serum gentamicin levels, 0.62 µg/mL on POD 3 and 0.41 µg/mL on day 10, fell well within this safety range (Table 3). Systemic therapy consisted of 72 hours of IV cefazolin (1 g every eight hours) followed by six weeks of oral cefaclor (250 mg three times daily), providing culture‑directed coverage for methicillin‑susceptible *Staphylococcus lugdunensis* while CLAP maintained a high intramedullary gradient; this local-systemic combination mirrors recent series [11, 19]. At a perfusate of 1.2 mg/mL (≈ 1,200 µg/mL), intramedullary concentrations exceed commonly reported staphylococcal biofilm‑eradication levels [19]. Because CLAP achieves local drug levels far above serum‑based breakpoints, CLSI/EUCAST interpretive categories do not predict locally perfused efficacy; clinical success has been reported even with gentamicin MICs up to 16 µg/mL [10, 11], and in our case, the local concentration‑to‑MIC ratio exceeded ~1,200:1.

Accounting for dilution: Even if intramedullary concentrations were reduced 10-fold by postoperative bleeding or exudate, continuous perfusion would still surpass the minimum biofilm‑eradication concentration (MBEC). Maruo et al. [11] documented a median of 502 µg/mL during 1.2 mg/mL perfusion; a tenfold dilution would yield ≈ 50 µg/mL-within published MBEC ranges for staphylococcal biofilms [19]. Concurrent NPWT continuously evacuates exudate and helps preserve this concentration gradient [10, 11, 17].

Similarly, PAT grafting is advantageous only in carefully selected wounds that already possess a robust, well‑vascularized granulation bed. Because PAT is a thin, pliable tissue sheet, it will not survive on poorly perfused, contaminated, or hardware‑exposed surfaces such as a freshly debrided medial tibial cortex. Critical limitations include minimal bulk provision, inability to obliterate deep dead space, and dependence on optimal wound‑bed preparation. Comparative analysis with alternatives reveals that muscle flaps (gastrocnemius, soleus) or free tissue transfer (anterolateral thigh, perforator‑based fasciocutaneous flaps) offer superior perfusion, dead‑space obliteration, and reliable coverage of exposed hardware, but require longer operative times, increased donor‑site morbidity, microsurgical expertise, and dependable recipient vessels, considerations that may be prohibitive in older adults. In our patient, 14 days of CLAP perfusion combined with NPWT produced a healthy granulation layer over the cortex, fulfilling the strict prerequisites for PAT acceptance. The graft was harvested through a small incision with negligible donor‑site morbidity [12, 13] and provided durable coverage that remained intact at six‑month follow‑up. Given the patient’s age, comorbidities, and limited recipient‑vessel options, PAT grafting offered a low‑risk alternative to muscle or free‑flap reconstruction, rather than a universal substitute for those techniques.

The exceptionally long latency between the initial envenomation and infection onset raises important questions about the underlying pathophysiology. *Staphylococcus lugdunensis*, isolated from debrided bone in this case, is a coagulase‑negative staphylococcus with virulence characteristics resembling *Staphylococcus* *aureus *[9]. It demonstrates osteotropism and the ability to persist intracellularly within osteoblasts, forming biofilms and evading immune surveillance, features that promote dormancy and delayed reactivation. Although chronic draining osteomyelitis can undergo malignant transformation into squamous‑cell carcinoma (Marjolin ulcer), deep‑margin biopsy and p40/p63/CK5/6 immunostaining in our patient showed only reactive pseudo‑epitheliomatous hyperplasia, conclusively ruling out carcinoma.

We hypothesize that bacterial inoculation occurred at the time of the original snakebite, facilitated by the exceptional fang length of the habu snake (15-20 mm) and venom enzymes such as metalloproteinases and hyaluronidases that enable deep‑tissue penetration [3, 4]. The anterior tibial crest, with minimal soft‑tissue coverage, would have been particularly vulnerable to direct periosteal or cortical inoculation. The absence of subsequent trauma and the precise anatomical correlation between the historical bite and current lesion further support this mechanism.

Although molecular strain typing was not performed, the combination of anatomical, microbiological, and imaging findings provides strong circumstantial evidence for dormant infection re‑emerging. Previous reports have described chronic osteomyelitis re‑emerging decades after presumed initial inoculation, particularly involving staphylococcal species [15].

Systemic inflammatory markers are often unhelpful once osteomyelitis becomes chronic. The infection is compartmentalized within necrotic bone and biofilm, so cytokine spillover is limited and acute‑phase reactants remain low. Panteli et al. found normal or only mildly raised CRP and WBC counts in nearly half of chronic cases, underscoring the poor sensitivity of these tests [8, 19]. Our patient’s CRP and WBC values (Table 1) mirror that experience and reinforce the need for imaging, histology, and culture to secure the diagnosis.

Our diagnostic approach highlights the utility of multimodal evaluation in older adults with chronic draining wounds. MRI with STIR sequences was essential in identifying intramedullary involvement and guiding iMAP pin placement. Tissue sampling focused on excluding malignancy, while definitive microbial identification provided therapeutic direction. This integrative strategy enabled a confident diagnosis without the need for extensive histopathological analysis, which may be contraindicated in frail patients.

This case offers important insights for clinicians managing chronic wounds and osteomyelitis in older adults. First, anatomical regions with minimal soft‑tissue coverage, such as the anterior tibial crest, are especially prone to deep bacterial inoculation during penetrating trauma. Second, snakebite‑related osteomyelitis should be considered in the differential diagnosis of chronic draining sinuses, even after exceptionally long latency periods. Third, CLAP and PAT grafting represent viable alternatives to traditional reconstruction in select high‑risk patients, offering effective infection control and durable soft‑tissue coverage with a reduced perioperative burden. Finally, these techniques are highly reproducible and adaptable for most tertiary‑care centers, requiring only standard instruments and widely available materials.

The described approach is both practical and accessible. CLAP can be implemented using iMAP pins and standard infusion pumps, while PAT graft harvesting can be performed via the same incision used for split‑thickness skin grafts, minimizing operative complexity. In this case, safety was ensured through routine monitoring protocols, including serial serum gentamicin levels and renal function tests (Table 3), which can be easily adopted in other institutions [10].

This case report has several limitations. Because the initial envenomation details are based on patient recollection, recall bias is possible. While the anatomical concordance of the current lesion with the historical bite site is compelling, a causal link cannot be definitively proven without molecular typing. The diagnosis was based on clinical, imaging, and microbiological correlation rather than molecular confirmation of strain identity, which could have definitively linked the current infection to the original inoculum. Additionally, as a single case report, generalizability is limited. Nonetheless, the reproducibility of the technique and the favorable outcome suggest potential applicability in similar clinical scenarios. Moreover, it provides proof‑of‑concept for a low‑morbidity treatment strategy combining local antibiotic delivery with simplified tissue reconstruction in older adults with chronic bone infections. Future investigations should explore larger case series, long‑term outcome comparisons with conventional methods, and cost‑effectiveness analyses. Expanded study of PAT graft integration across various wound types may also broaden its utility in reconstructive surgery.

## Conclusions

In this older adult with chronic tibial osteomyelitis, targeted minimal debridement followed by the CLAP drug‑delivery system and subsequent PAT grafting achieved infection control and durable soft‑tissue coverage with favorable functional recovery. This case supports combining high‑concentration local antibiotic delivery with low‑morbidity reconstruction in carefully selected patients, those with a well‑vascularized granulation bed, limited dead space, and no exposed hardware.

The CLAP delivery system should be used as an adjunct to surgical source control rather than as a standalone therapy approach. PAT is not a substitute for muscle or free flaps when bulk transfer or dead‑space obliteration is required. The exceptionally long 37‑year latency after habu envenomation highlights the potential for markedly delayed infectious sequelae following penetrating injury. Larger case series are needed to refine patient‑selection criteria, compare outcomes with conventional reconstruction, and assess cost‑effectiveness.
